# Dielectric Relaxation Spectroscopy and Synergy Effects in Epoxy/MWCNT/Ni@C Composites

**DOI:** 10.3390/nano11020555

**Published:** 2021-02-23

**Authors:** Darya Meisak, Jan Macutkevic, Algirdas Selskis, Polina Kuzhir, Juras Banys

**Affiliations:** 1 Physics Faculty, Vilnius University, Sauletekio Avenue 3, LT-10222 Vilnius, Lithuania; dariameysak@gmail.com (D.M.); juras.banys@ff.vu.lt (J.B.); 2 Institute for Nuclear Problems, Belarusian State University, Bobruiskaya Street 11, 220006 Minsk, Belarus; polina.kuzhir@uef.fi; 3 Center for Physical Science and Technology, Sauletekio Avenue 3, LT-10222 Vilnius, Lithuania; algirdas.selskis@ftmc.lt; 4 Department of Physics and Mathematics, Institute of Photonics, University of Eastern Finland, Yliopistokatu 7, FI-80101 Joensuu, Finland

**Keywords:** carbon nanotubes, carbon-coated Ni, epoxy, electrical properties, conductivity, relaxation time

## Abstract

The dielectric/electric properties of the Ni@C (carbon-coated Ni)/epoxy composites and Ni@C/MWCNTs (multi-walled carbon nanotubes)/epoxy composites loaded with fixed MWCNTs amount just below the percolation threshold (0.09 vol.%) and Ni@C at different concentrations up to 1 vol.% were investigated in broad frequency (20 Hz–40 GHz) and temperature (30 K–500 K) regions. In composites with the only Ni@C nanoparticles, the electrical percolation threshold was determined between 10 and 15 vol.%. Above the percolation threshold the dielectric permittivity (ε’) and the electrical conductivity (σ) of the composites loaded with Ni@C only are high enough, i.e., ε’ = 10^5^ and σ = 0.6 S/m at 100 Hz for composites with 30 vol.% Ni@C, to be used for electromagnetic shielding applications. The annealing to 500 K was proved to be an effective and simple tool to decrease the percolation threshold in epoxy/Ni@C composites. For hybrid composites series an optimal concentration of Ni@C (0.2 vol.%) was determined, leading to the conductivity absolute values several orders of magnitude higher than that of a composite filled with MWCNTs only. The synergy effects of using both fillers have been discussed. Below room temperature the electrical transport is mainly governed by epoxy resin compression in all composites, while the electron tunnelling was observed only in hybrid composites below 200 K. At higher temperatures (above 400 K), in addition to the nanoparticles redistribution effects, the electrical conductivity of epoxy resin makes a significant contribution to the total composite conductivity. The dielectric relaxation spectroscopy allows estimating the nanoparticles distributions in polymer matrix and could be used as the non-destructive and fast alternate to microscopy techniques for general polymer composite fabrication control.

## 1. Introduction

Polymer composites loaded with carbon nanoparticles have attracted superior attention due to the variety of their electric, mechanical and thermal properties, wide range of their functionalities and multifunctionalities, followed by the possibility of fine tuning/control of their specific features by playing with the fillers concentrations, fabrication methods, matrices origins and applying external forces [1,2]. The high aspect ratio carbon nanotubes (CNTs) give conductive properties to the insulating matrices at their sufficiently low content up to 1–2 wt.% [3]. However, huge Van der Waals forces between individual CNTs lead to their agglomeration, which increases the percolation threshold (some critical concentration at which Direct Current (DC) conductivity occurs [4]). Rising the CNTs concentration not only makes the composite manufacturing process more laborious, but also might impair the mechanical properties of the resultant polymer product. Moreover, CNTs are expensive and might by toxic [5]. Therefore, reducing the electrical percolation threshold along with simultaneous maintaining the optimal mechanical properties of the polymer composites at as low as possible amount of expensive and toxic filler is still an actual problem.

One of the possible solutions is the development of hybrid multifunctional composite materials. Often, due to the proper distribution of few different fillers in the matrix and their simultaneous participation in the percolation network formation, synergy effects can occur. Mostly, the electrical conductivity improvement of a multiphase composite is accompanied by a decrease in the percolation threshold compared to single-filler composites. A considerable number of papers have been published with successful detection of synergy effects of different carbon allotropes [6,7,8,9]. Composites with the combination of particles of different natures (carbon and non-carbon) can have optimal dielectric and magnetic properties [10,11,12,13,14]. In particular, the introduction of cobalt nanoparticles inside MWCNTs led not only to a substantial increase electrical conductivity, but also stimulated the emergence of ferromagnetism as well [15]. Such multiphase systems are especially interesting for solving the problems of electromagnetic compatibility, since the regulation of the dissimilar particles ratio leads to a variation of dielectric and magnetic losses with the electromagnetic response control [16,17].

Nickel (Ni) nanosized particles and carbon-coated Ni nanoparticles (Ni@C) have superparamagnetic or ferromagnetic features, which, due to the presence of magnetic losses [18,19], make them promising for the design of microwave absorbing devices. The thermal behaviour of broadband dielectric and magnetic properties has been studied for the composites made of polymer filled with Ni@C concentrations below the percolation threshold [20]. However, the dielectric properties of composites with Ni@C content above the percolation threshold, the electrical percolation in these composites, and hybrid composites combining Ni@C and MWCNTs fillers have not been studied yet. The problem is relevant also due to the fact that the electrical percolation threshold in composites with quasi-spherical particles can be very low, substantially lower than predicted by the excluded volume theory, so that these composites can substitute CNT based composites in various electronics applications [21,22].

In this work we study epoxy resin composites filled with Ni@C at various volume contents in order to determine the electrical percolation and broadband dielectric/electric properties of these composites in a wide temperature range. Moreover, using the dielectric spectroscopy methods we investigate the properties of hybrid Ni@C/MWCNTs/epoxy resin composites materials in order to discover possible synergy between constituent components.

## 2. Materials and Methods

To prepare composites commercial epoxy resin Epikote 828 with density of 1.16 g/cm^3^ was used as the polymer matrix. Multi-walled carbon nanotubes and Ni nanoparticles covered with carbon (Ni@C) were used as fillers. MWCNTs grown by the chemical vapor deposition method [23] had the average outer diameter of 20–40 nm, the length of 0.5–200 µm and the density of 2.0 g/cm^3^. Commercially available 20 nm-thick Ni nanoparticles with density of 8.9 g/cm^3^ were used for composite preparation [24]. Ni nanoparticles were coated with several closely compacted carbon layers being a few nanometers thick [25,26].

Both series, mono-filled (Ni@C/epoxy) and hybrid (Ni@C/MWCNTs/epoxy) composites, were prepared according to the standard procedure for filler particles dispersing in an epoxy matrix [27,28]. The producing procedure of Ni@C/epoxy composites with Ni@C concentrations of 10, 15, 25 and 30 vol.% included the dispersion of Ni@C particles in isopropanol using an ultrasonic probe for 1 h, repeated one-hour sonication after addition of epoxy resin to the Ni@C/isopropanol suspension and curing after isopropanol evaporation. The ultrasonic probe power was 10 W, the oscillation amplitude was 25 μm and water cooling was done during the process. The procedure of hybrid Ni@C/MWCNTs/epoxy composites fabrication was more complex and time-consuming due to the separate dispersion of each filler in isopropanol by using ultrasonication (1-h probe for Ni@C/isopropanol, 1-h probe and 1-h bath for MWCNTs/isopropanol). Epoxy resin was added to MWCNTs/isopropanol suspension and the resulting mixture was subjected to re-sonication during 1 h. Using the fixed epoxy resin mass of 5 g for each sample, the volume concentrations of both fillers were estimated from weight concentrations taking into account the materials density mentioned above. The final 1 h ultrasonic treatment took place after mixing together both the Ni@C/isopropanol and MWCNTs/epoxy/isopropanol suspensions. The final stages were isopropanol evaporation and curing. The curing process of both composites series was carried out by triethylenetramine (TETA) hardener for 24 h at room temperature and 2 h at 100 °C in the oven. In both mono-filled and hybrid composites manufacturing techniques the hardener was added in a ratio of 1:10 with respect to the epoxy resin.

The techniques described above allowed us to obtain mono-filled Ni@C/epoxy composites with Ni@C concentration of 10, 15, 25 and 30 vol.%, as well as hybrid Ni@C/MWCNTs/epoxy composites with a fixed MWCNTs concentration of 0.09 vol.% (just below the percolation state in corresponding mono-filled composites [27]) and various Ni@C content of 0, 0.025, 0.2, 0.6, and 1 vol.%. Hybrid composites with relatively low Ni@C concentrations were prepared because composites with higher concentrations demonstrate non-uniform distributions of nanoparticles (will be shown below).

The complex dielectric permittivity was determined using an LCR meter HP4284A in the frequency range 20 Hz–1 MHz. The method is based on the use of equivalent electrical circuits. Selecting one of the allowed measuring capacitance C and loss tangent δ (the ratio of imaginary and real parts of complex dielectric permittivity) connected in parallel, the complex dielectric permittivity can be calculated from the planar capacitor equation [29]:

$$\varepsilon \prime =\frac{\left(C-C_{0}\right)d}{\varepsilon_{0}S}+1,$$

(1) $$tg\delta =\frac{Ctg\delta -C_{0}tg\delta_{0}}{C-C_{0}}$$

where *C* and *tgδ* are capacitance and tangent of losses of the systems with the sample, *C*_0_ and *tgδ*_0_ are capacitance and tangent of losses of the systems without the sample, *d* is height of the sample, *S* is the area of the sample, *ε*_0_ is the dielectric permittivity of vacuum. The electrical conductivity σ’ was therefore calculated as [29]:

σ’ = 2πνε’tgδε_0_ (2)

where π is the pi constant and ν is the frequency. For temperature measurements, the home-made furnace (300–500 K) and the closed cycle helium cryostat (300–30 K) were used. The silver paste was used for contacts. The dielectric measurements in the 1 MHz-3 GHz frequency range were performed with the coaxial line method using a vector network analyser Agilent 8714ET, by measuring the complex reflection coefficient from the coaxial line terminated by short-circuit connected sample. The detailed description of this method can be found in literature [29]. The thin-rod method in the waveguide [29] was used for microwave measurements in the frequency range from 27 to 39 GHz. In more details, rod-like samples having a diameter of 1 mm, were used and placed at the centre of the waveguide with their axis parallel to the electric field vector. The scalar reflection and transmission coefficients were measured using Elmika scalar network analyser R2400. All samples were glued with silver paint to the sample holder. The dielectric properties of samples were obtained by application of a modified Newton optimization algorithm based on the waveguide formalism [29]. For each concentration up to 10 samples were tested under the same conditions, then the average values were calculated and they are in the present study.

Structure and morphology properties of the samples were studied by scanning electron microscopy (SEM) using a Helios NanoLab 650 microscope (Thermofisher Scientific, Hillsboro, OR, USA).

Thermogravimetric measurements were carried out using an analyser STA 6000 (Perkin Elmer). The samples were ground into powder; 35–45 mg of the studied materials were measured in a corundum platter with the heating rate of 10 K/min in air.

## 3. Results

### 3.1. Sample Characterization

**Figure 1:**
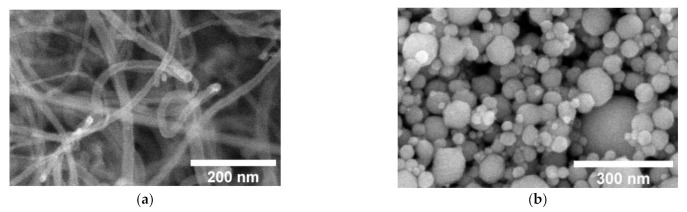
Scanning electron microscopy micrographs of MWCNTs (**a**) and Ni@C (carbon-coated Ni) (**b**) nanoparticles.

SEM analysis was performed to get information about the microstructure of nanofillers used, as well as about their dispersion in the epoxy matrix. Figure 1a,b shows the microstructure of MWCNTs and Ni@C nanoparticles.

**Figure 2:**
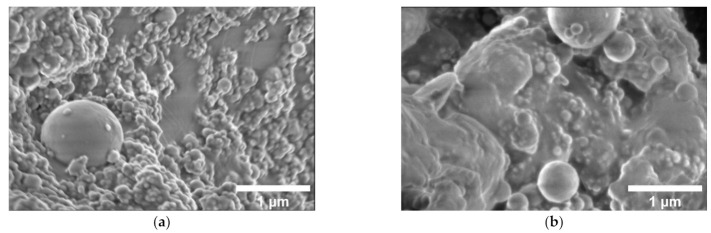
Scanning electron microscopy micrographs of mono-filled Ni@C/epoxy resin composites with (**a**) 10 and (**b**) 25 vol.% of Ni@C.

**Figure 3:**
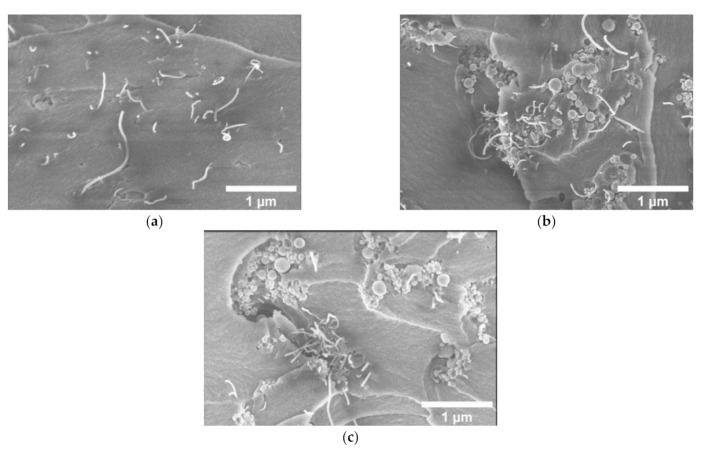
Scanning electron microscopy micrographs of hybrid Ni@C/MWCNTs/epoxy resin composites with 0.09 vol.% of MWCNTs and (**a**) 0, (**b**) 0.6 and (**c**) 1 vol.% of Ni@C.

The SEM images of both mono-filled Ni@C/epoxy and hybrid Ni@C/MWCNTs/epoxy composite materials series are presented in Figure 2a and Figure 3a–c, respectively. As it can be seen from the Figure 2a,b, the composites filled with the lowest Ni@C content (10 vol.%) are characterized by a good distribution of nanoparticles, while high (25 vol.%) loading leads to a lower quality dispersion, which manifests in the presence of larger number of Ni@C agglomerates of different sizes. The MWCNTs distribution in the composite without Ni@C is good (see Figure 3a). However, Figure 3b,c clearly show that an increase of Ni@C concentration from 0.6 to 1 vol.% in the hybrid composites is accompanied by a MWCNTs distribution deterioration (an increase of the agglomerates number).

**Figure 4:**
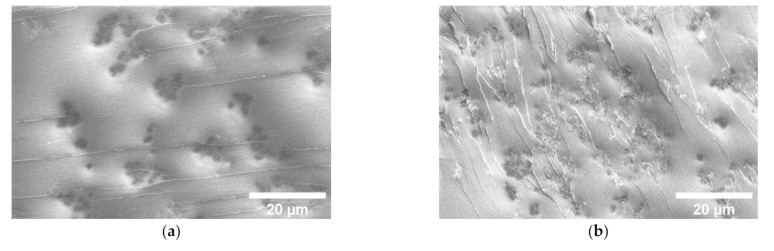

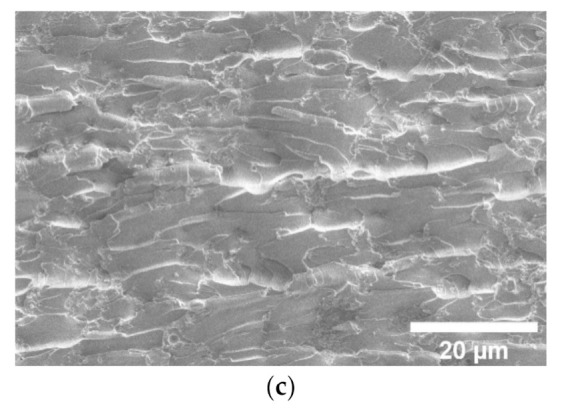
Panoramic scanning electron microscopy micrographs of hybrid Ni@C/MWCNTs/epoxy resin composites with 0.09 vol.% of MWCNTs and (**a**) 0, (**b**) 0.6 and (**c**) 1 vol.% of Ni@C.

In order to see the macroscopic distribution of MWCNTs, the panoramic SEM of composites is presented in Figure 4a–c (MWCNT clusters are observed as black spots, which are confirmed by higher resolution SEM pictures). The MWCNT network is clearly observed in composites with 0.6 vol.% Ni@C (Figure 4), in composites with 1 vol.% clusters of MWCNT are uniformly distributed, while in composites without Ni@C, no macroscopic structure of the MWCNT is observed. This is in good agreement with previously reported results that the MWCNT clustering can decrease the percolation threshold value [4]. Smaller Ni@C clusters acts as separators of MWCNT clusters (Figure 3b) and support certain macroscopic structures of the MWCNT network (Figure 4b,c).

**Figure 5:**
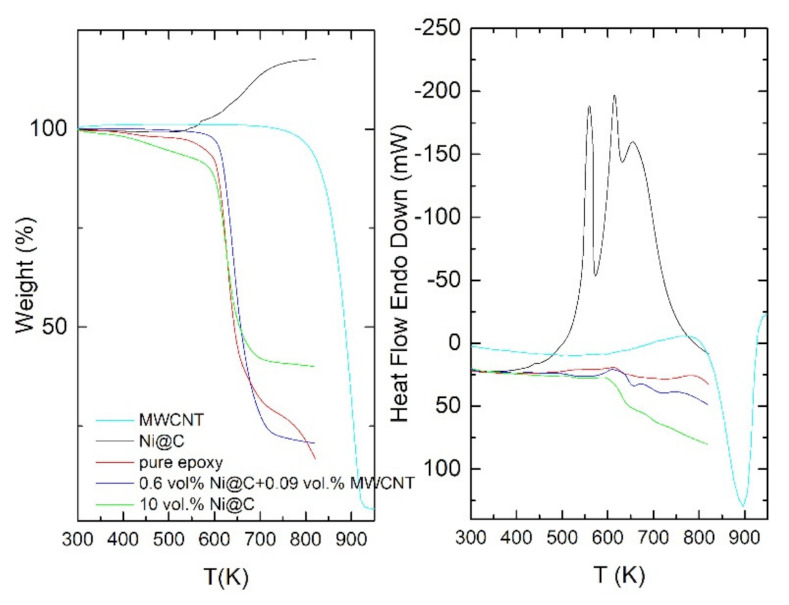
Thermogravimetric analysis of MWCNT, Ni@C nanoparticles, epoxy and composites with these inclusions.

Results of thermogravimetric analysis of MWCNT, Ni@C nanoparticles, epoxy and composites with these inclusions are presented in Figure 5. The small weight decrease in the temperature range 300–600 K (the biggest decrease in weight is observed for composite with 0.6 vol.% Ni@C + 0.09 vol.% MWCNT, however it is only 5%) can be explained by the decrease of humidity. Therefore, it is possible to conclude that powders and composites are stable up to 600 K. Thus, namely this temperature range was selected for broadband investigations. The oxidation processes in powders, epoxy and composites starts at higher temperatures (above 600 K) and is followed by the significant decrease in weight.

### 3.2. Dielectric/Electric Properties of Mono-Filled Ni@C/Epoxy Composites

#### 3.2.1. Room-Temperature Region

**Figure 6:**
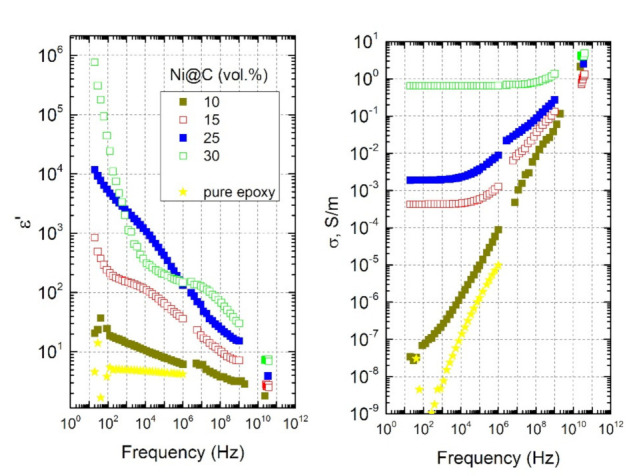
Broadband frequency dependencies of the real part of dielectric permittivity and the electrical conductivity of mono-filled Ni@C/epoxy composites with different Ni@C content at room temperature.

The frequency dependencies of dielectric permittivity (ε’) and the electrical conductivity (σ) of mono-filled Ni@C/epoxy composite materials at room temperature are presented in Figure 6.

For the sample with the lowest Ni@C concentration of 10 vol.% (dark green close symbols), the dielectric permittivity is weakly dependent on the frequency, and the DC conductivity plateau is absent (similarly to empty epoxy resin [30]). Meanwhile, the rest of the samples demonstrate a pronounced conductive behaviour, namely, the presence of a strong ε’ frequency dependence and the DC conductivity plateau (frequency-independent part of σ). With increasing of embedded Ni@C nanoparticle concentration, the DC conductivity absolute value increases. Such result indicates that the percolation threshold of mono-filled Ni@C/epoxy composites is in the range between 10 and 15 vol.% of Ni@C nanoparticles.

Moreover, above the percolation threshold the dielectric permittivity (ε’) and the electrical conductivity (σ) of composites with single Ni@C inclusions are very high (ε’ = 10^5^ and σ = 0.6 S/m at 100 Hz for composites with 30 vol.% Ni@C). The dielectric permittivity strongly decreases, while the electrical conductivity strongly increases with frequency. However, the complex dielectric permittivity remains quite high even in microwave frequency range, so that these composites are suitable for electromagnetic shielding applications.

#### 3.2.2. Temperature-Dependent Properties and Relaxation Time Distributions

**Figure 7:**
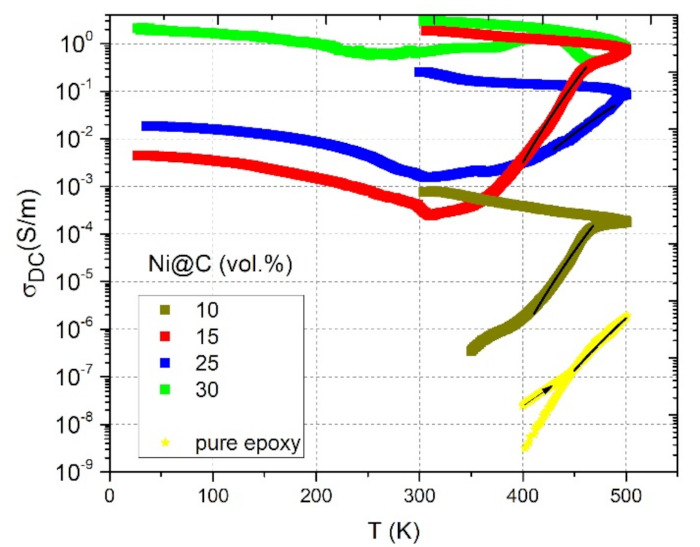
Temperature dependence of the DC conductivity of mono-filled Ni@C/epoxy composites with different Ni@C content. Solid lines at high temperatures correspond to approximations according to Equation (3).

The temperature dependencies of DC conductivity in a wide temperature range for mono-filled Ni@C/epoxy composite materials are presented in Figure 7.

For non-conductive at room temperature composite with 10 vol.% Ni@C content, DC conductivity appeared at temperatures above 350 K, coursed by the epoxy resin conductivity contribution (at high temperatures epoxy resin becomes conductive) [31]. For the conductive at room temperature samples, a small narrow-temperature (from room temperature to 310 K) reduction in DC conductivity is observed, which is obviously associated with polymer matrix thermal expansion. Then, further heating up to the maximum temperature (500 K) was accompanied by a monotonic increase in DC conductivity values by 2–3 orders of magnitude, which is related to the epoxy resin conductivity contribution. The second process affecting the increase of $\sigma_{DC}$ is nanoparticles redistribution inside the matrix (large agglomerates break down into smaller ones) [31]. That is why, after annealing, there is such pronounced hysteresis of DC conductivity for all samples, which intensifies even more on the back way (from 500 K to room temperature) during cooling due to the compression of the polymer matrix. Moreover, after annealing the DC conductivity of Ni@C 15 vol.%/epoxy became higher than for 25 vol.%, which indicates a more successful nanoparticles redistribution in the composite with a lower filler content due to initially smaller agglomerates number and their size. The composite with 10 vol.% Ni@C remains conductive after annealing. It means that annealing can be effectively used to decrease the percolation threshold in epoxy/Ni@C composites. The lowest increase of electrical conductivity after annealing is observed for composites with the highest Ni@C concentration obviously due the lowest distances between conductive clusters and the most stable initial percolation network formed before thermal treatment.

Cooling of unannealed samples from room temperature to 30 K is characterized by a monotonic increase in DC conductivity. A possible explanation is the epoxy resin compression during cooling, which reduces the distance between the nanoparticles and, as a result, contributes to an increase in conductivity.

In the heating mode, the DC conductivity can be fitted by the Arrhenius law:

(3) $$\sigma_{DC}=\sigma_{0}e^{-\frac{E_{A}}{k_{B}T}},$$

where $\sigma_{0}$ is the preexponential factor, $k_{B}$ is the Boltzmann constant, and $E_{A}$ is the activation energy. The Arrhenius behaviour is typical for the thermally activated conductivity, observed, e. g. in pure epoxy resin [27]. Obtained parameters are presented in Table 1. The following observation can be made: the higher Ni@C concentration (and, consequently, the initial room temperature DC conductivity), the lower the activation energy (Table 1). This effect can be explained by the lower impact of the epoxy resin conductivity on the resultant composite conductivity at higher nanoparticles concentrations.

The procedure used for calculating the relaxation time distribution is described in detail in [32,33]. In short, it consists in solving an integral equation with known frequency dependences of the complex impedance *Z*:

(4) $$Z\left(\nu \right)=Z_{\infty }+\Delta Z\int_{-\infty }^{\infty }\frac{f\left(\tau \right)d\log\tau }{1+i\omega \tau },$$

where *f*(*τ*) is the relaxation time distribution function.

The complex impedance ($Z=Z^{\prime }-iZ^{\prime \prime }$) can be obtained from the experimental data of the complex dielectric permittivity by the following expressions:

(5) $$Z^{\prime }=\frac{\varepsilon^{\prime \prime }}{{\varepsilon^{\prime }}^{2}+{\varepsilon^{\prime \prime }}^{2}}\frac{1}{2\pi \nu \varepsilon_{0}}.$$

(6) $$Z^{\prime \prime }=\frac{\varepsilon^{\prime }}{{\varepsilon^{\prime }}^{2}+{\varepsilon^{\prime \prime }}^{2}}\frac{1}{2\pi \nu \varepsilon_{0}}.$$

**Figure 8:**
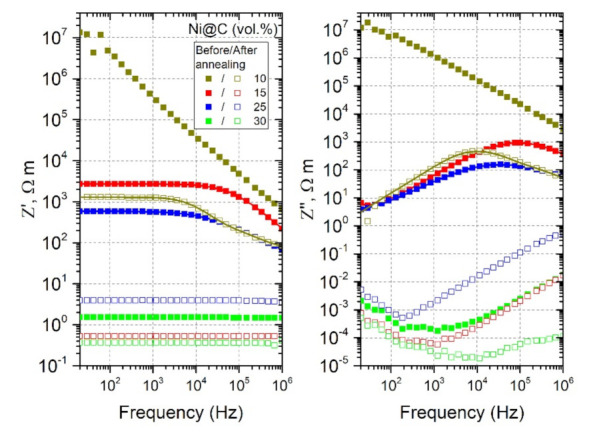
Frequency dependence of the complex impedance (calculated according to Equations (5) and (6)) for mono-filled Ni@C/epoxy composites at room temperature before and after annealing at 500 K.

The calculated frequency dependences of complex impedance for all mono-filled Ni@C/epoxy composites under study before and after annealing at 500 K are presented in Figure 8.

The frequency-independent plateau of $Z^{\prime }$ disappears and $Z^{\prime \prime }$ has a maximum close to the critical frequency (frequency at which the conductivity starts increase from their DC conductivity plateau). The critical frequency position determines the relaxation time (the position of the relaxation time distribution maximum). For this reason, if the critical frequency is outside the investigated experimental frequency range, then it is not possible to determine quantitatively the relaxation time distribution. Thus, analysing Figure 8, one can conclude that, among the studied samples, only for three of them, the relaxation time distributions are available from the collected experimental data: 15, 25 vol.% Ni@C before annealing and 10 vol.% Ni@C after annealing.

**Figure 9:**
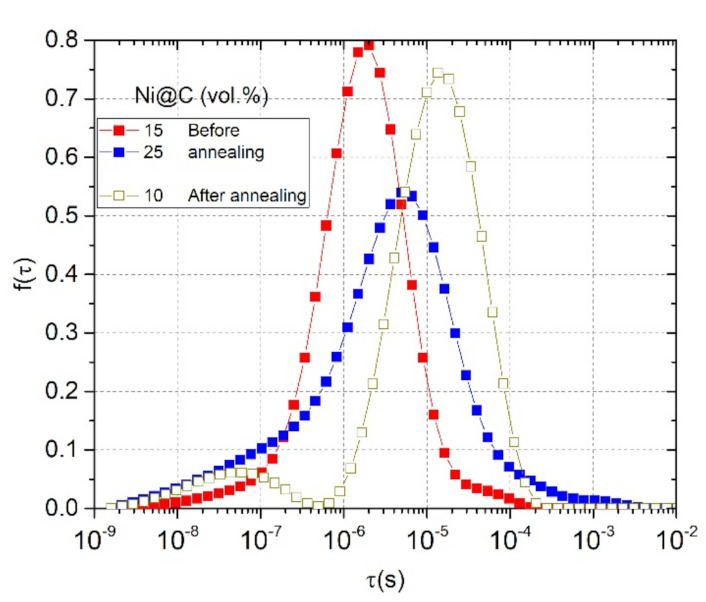
Relaxation time distributions (calculated according to Equation (4)) for mono-filled Ni@C/epoxy composites before and after annealing at 500 K.

The obtained distributions of relaxation times are presented in Figure 9. Considering the interpretation of distribution of relaxation times in percolative composites, the relaxation time τ = RC = C/σ, where C is the capacitance of one cluster of conductive nanoparticles, R is the resistivity and σ is the conductivity inside one clusters or between neighbouring clusters. The capacitance of clusters is dependent only on geometrical parameters of clusters, for example if we assume spherical clusters, their capacitance is

C = 4πε_0_r, (7)

where r is the effective radius of clusters. The tunnelling conductivity between clusters is also dependent on nanoparticles distribution inside the polymer matrix. Thus, short relaxation times in distributions correspond to the relaxation in small Ni@C clusters where they are distributed more homogeneously and long relaxation times correspond to large clusters of nanoparticles. From the distribution of relaxation times, it is difficult to speak about the real Ni@C geometrical shape distribution because the conductivity σ is also dependent on the Ni@C concentration (Figure 6) and on the potential barrier for electrons tunnelling between clusters. Therefore, if the distribution of conductive nanoparticles is the same, the distribution of relaxation times should be observed at shortest relaxation times for composites with higher nanoparticles concentrations. In Figure 9 the opposite situation is observed, the distribution of relaxation times of composites with 15 vol.% Ni@C is observed at shorter relaxation times in comparison with distributions of relaxation times of composites with 25 vol.% Ni@C. This indicates a better distribution of Ni@C nanoparticles in composites with smaller Ni@C concentrations in good agreement with SEM investigations (Figure 2).

The relaxation time distributions show a clear deviation from the classical percolation theory: a more conductive sample (25 vol.%) has a lower critical frequency (Figure 6) and, consequently, a longer relaxation time. This is due to non-optimal nanoparticles distribution in the high-content sample (presence of large agglomerates in the Figure 2b). After annealing, this effect is enhanced: the DC conductivity of 15 vol.% Ni@C/epoxy is higher than that of 25 vol.% Ni@C/epoxy. Thus, the physics coming from the analysis of dielectric spectra, in particular the dielectric relaxation spectroscopy, allows to estimate the nanoparticle distribution level in the polymer matrix.

**Figure 10:**
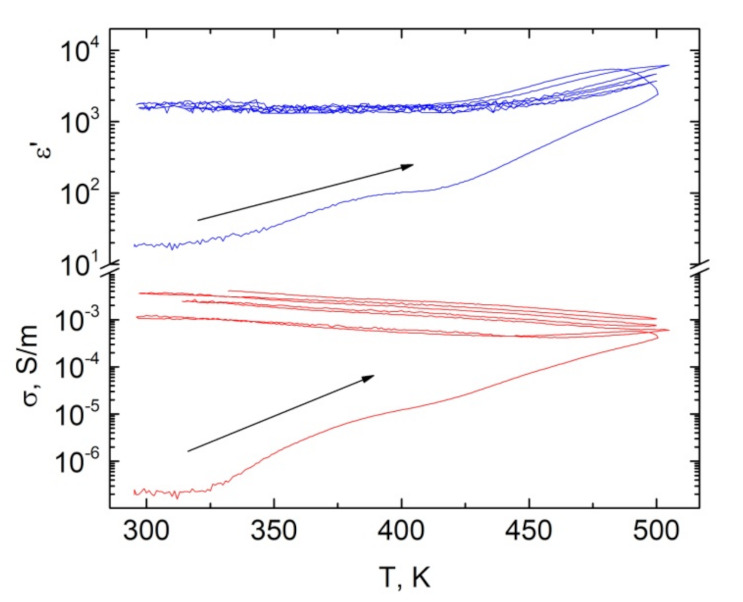
Temperature dependence of dielectric permittivity and electrical conductivity selected at 129 Hz for composite filled with 10 vol% Ni@C inclusions (multiple heating/cooling cycles).

In order to study the hysteresis of electrical properties in heating/cooling in details the experiment was performed with multiple (four) heating/cooling (from room temperature up to 500 K and down to room temperature) cycles (Figure 10). It is clearly observed that the biggest differences in heating and cooling regime are observed in the first cycle, while in the next cycles the hysteresis is less pronounced. Nevertheless, the electrical conductivity still increases after each heating/cooling cycle, while the dielectric permittivity remains almost stable. It denotes that impedance spectra and distributions of relaxation times are not sustainable in all heating/cooling cycles [28].

The temperature behaviour of electrical properties of epoxy resin composites can be also related with the post curing effects [34]. Prepared composites were cured at 100 °C (please see part 2). However, their electrical properties keep non-persistent even in multiple heating/cooling regime, therefore broadband properties of fabricated composites are mainly related to positive and negative temperature coefficients effects of resistivity [28,30,31].

### 3.3. Hybrid Ni@C/MWCNTs/Epoxy Composites

#### 3.3.1. Room-Temperature Properties

**Figure 11:**
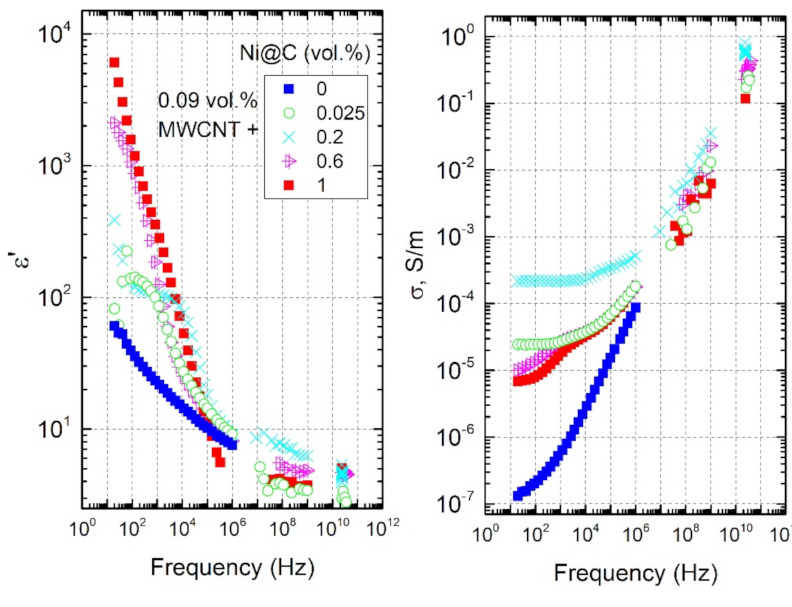
Broadband frequency dependencies of the dielectric permittivity and the electrical conductivity of hybrid Ni@C/MWCNTs/epoxy composites with fixed MWCNTs concentration of 0.09 vol.% and different Ni@C content at room temperature.

**Figure 12:**
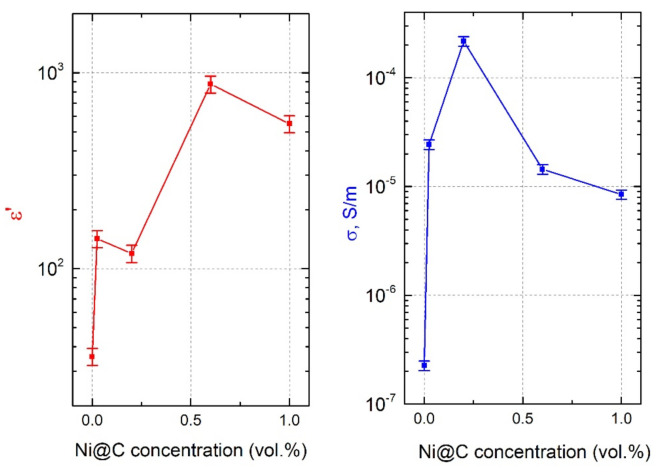
Ni@C-concentration dependencies of the dielectric permittivity and the electrical conductivity for composites with fixed MWCNTs content of 0.09 vol.% at room temperature and frequency of 129 Hz.

The frequency and concentration dependencies of ε’ and σ for hybrid Ni@C/MWCNTs/epoxy composite materials at room temperature are presented in Figure 11 and Figure 12, respectively.

One can see that the addition of Ni@C to initially non-conductive composite with MWCNTs (a pre-percolation state) causes the appearance of the DC conductivity. The DC conductivity changes in a non-monotonically manner with increasing of Ni@C concentration. First, the $\sigma_{DC}$ increases, then, having reached a maximum at the Ni@C concentration of 0.2 vol.%, it begins decreasing. This indicates the synergy effect between two fillers at low Ni@C content. Perhaps small Ni@C clusters, located in between the nanotubes, help them to complete the formation of the MWCNTs percolation network. Small Ni@C amounts up to 0.2 vol.% improve the MWCNTs dispersion in the polymer matrix, while at higher Ni@C concentrations an increase of the agglomerates number and, as a consequence, a MWCNTs distribution deterioration is observed (see SEM-images in Figure 3).

#### 3.3.2. Temperature-Dependent Properties and Relaxation Time Distributions

**Figure 13:**
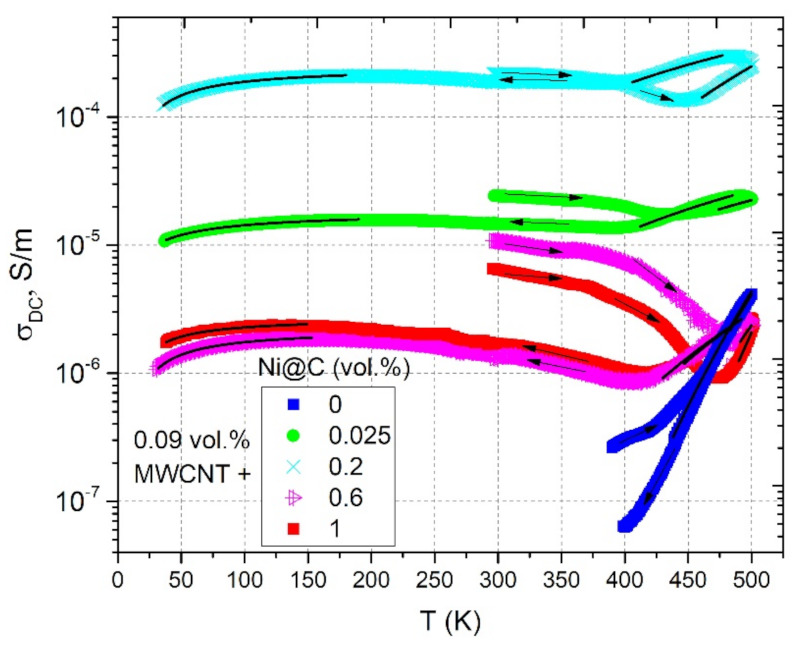
Temperature dependence of the DC conductivity of hybrid Ni@C/MWCNTs/epoxy composites with fixed MWCNTs concentration of 0.09 vol.% and different Ni@C content. Solid lines at high and low temperatures correspond to approximations according to Equations (3) and (8), respectively.

The temperature dependencies of DC conductivity in a wide temperature range for hybrid Ni@C/MWCNTs/epoxy composite materials are presented in Figure 13.

In the sample without Ni@C the DC conductivity is observed only at temperatures above 400 K due to the epoxy resin contribution. All composites with Ni@C have qualitatively similar temperature dependencies of $\sigma_{DC}$. The temperature sections from room temperature to 500 K and from 500 K to room temperature have the character that was observed for the heating area in the mono-filled Ni@C/epoxy samples series. The only difference is the temperature intervals of each separate zone (see Figure 13 for more details). All samples from hybrid series are also characterized by hysteresis, but this is accompanied by a decrease of DC conductivity after annealing at room temperature. This may be due to the partial destruction of the percolation network after annealing. At high temperatures, the $\sigma_{DC}$ before and after annealing is well fitted by the Arrhenius law according to the Equation (3) with approximation parameters presented in the Table 2. Before annealing, the minimum activation energy is characteristic for the sample with the lowest Ni@C concentration (0.025 vol.%). After annealing, the activation energy completely correlates with the DC conductivity values: the higher $\sigma_{DC}$, the lower $E_{A}$. In this case the minimum activation energy is observed for the most conductive sample of 0.2 vol.% Ni@C. Similarly, as in the case of Ni@C/epoxy resin composites the lowest activation energy indicates the lowest contribution of polymer matrix conductivity to the total conductivity of composite, because the electrical conductivity of the percolation network is much bigger.

Cooling of the annealed samples from room temperature to 30 K is characterized by a monotonic DC conductivity decrease. On the low-temperature region, the $\sigma_{DC}$ is fitted well according to the tunnelling model [35]:

(8) $$\sigma_{DC}=\sigma_{0}e^{-\frac{T_{1}}{T+T_{0}}},$$

where $\sigma_{0}$ is the pre-exponential factor, $T_{1}$ is the energy required for an electron to cross the insulator gap between the conductive particle aggregates, and $T_{0}$ is the temperature above which thermally activated conduction over the barriers occurs.

Parameters $T_{1}$ and $T_{0}$ are determined by following expressions:

(9) $$T_{1}=\frac{wA\beta_{0}}{8\pi k_{B}},$$

(10) $$T_{0}=\frac{2T_{1}}{\pi \chi w},$$

where $\chi =\sqrt{2mV_{0}}/\hbar$ and $\beta_{0}=4V_{0}/ew$; m and e are the electron mass and charge, respectively;$\;V_{0}$ is the potential barrier amplitude; w the inter-particles distance (gap width); A is the area of capacitance formed by the junction; ħ is the Dirac constant. Obtained parameters are presented in Table 3. The ratio $T_{1}/T_{0}$ is proportional to the gap width w and the potential barrier $V_{0}$ amplitude. The minimum of $T_{1}/T_{0}$ is observed at 0.025 vol.% Ni@C, which is close to the optimal concentration (0.2 vol.% Ni@C) for electrical properties. Thus, at low temperatures, the main transport mechanism is electron tunnelling through the potential barrier. Obviously, the tunnelling conductivity is the typical electrical transport mechanism also in mono-filled Ni@C/epoxy resin composites. However, due to the small size of Ni@C particles the value of T_1_ is very low and the tunnelling conductivity could be observed at very low temperatures (lower than that available by our experimental technique).

**Figure 14:**
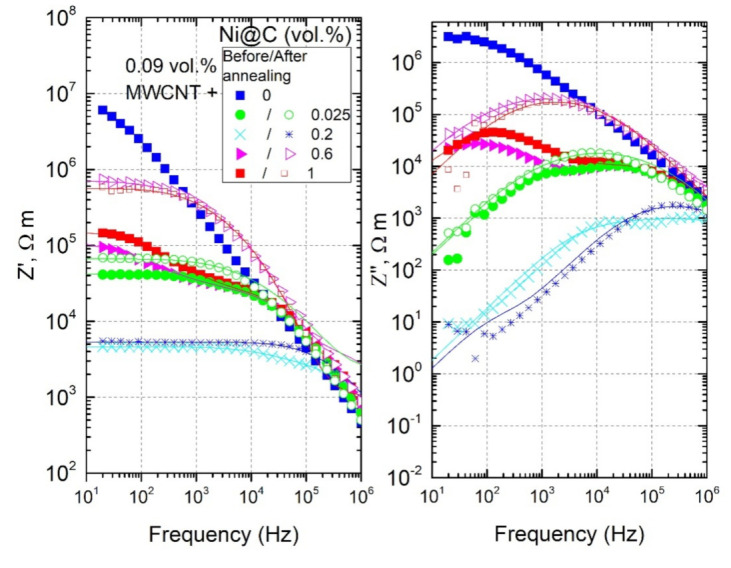
Frequency dependence of the complex impedance for hybrid Ni@C/MWCNTs/epoxy composites at room temperature before and after annealing at 500 K.

**Figure 15:**
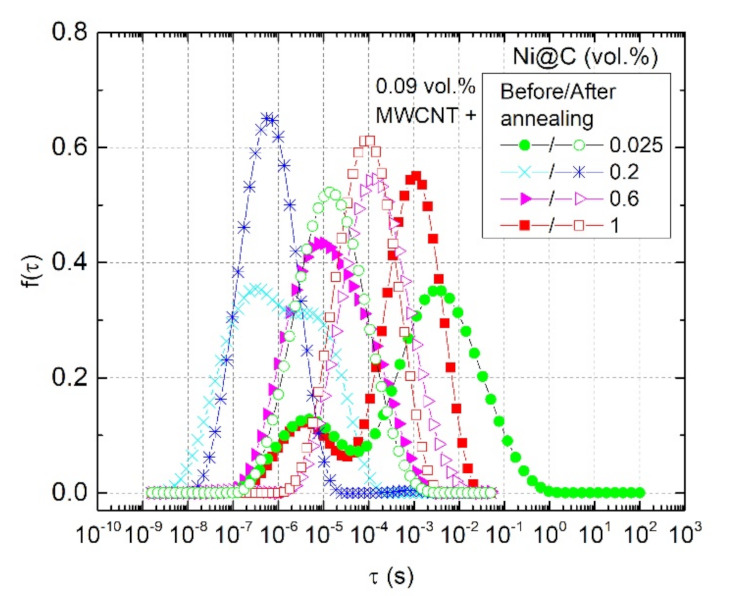
Relaxation time distributions for hybrid Ni@C/MWCNTs/epoxy composites before and after annealing at 500 K.

The frequency dependences of complex impedance and relaxation time distributions for all hybrid Ni@C/MWCNTs/epoxy composites before and after annealing at 500 K are presented in Figure 14 and Figure 15, respectively. Before annealing, the relaxation time distributions are not symmetric; however, one should trust the short-time maxima, because the additional maximum at longer relaxation times is related to nonohmic contacts. After annealing, the relaxation time distributions become symmetric for all composites, and the maxima of $f\left(\tau \right)$ shifts towards longer relaxation times, which corresponds to the conductivity decrease. The shortest relaxation time is observed for the sample with the best Ni@C nanoparticles distribution (0.2 vol.%). Moreover, for this sample the DC conductivity (and, consequently, the nanoparticles distribution) almost does not change after annealing (see Figure 13), while for the rest of the samples the $\sigma_{DC}$ deteriorates significantly.

## 4. Discussion and Conclusions

The dielectric/electric properties of the mono-filled Ni@C/epoxy composites with high volume Ni@C content (up to 30 vol.%) and hybrid Ni@C/MWCNTs/epoxy composites with fixed MWCNTs amount (just below percolation threshold in corresponding single-phase composites) and different Ni@C concentrations up to 1 vol.% were investigated in the broad frequency (20 Hz–40 GHz) and temperature (30 K–500 K) ranges. For mono-filled Ni@C/epoxy composites series, it was possible to roughly estimate the percolation threshold, which is in the range between 10 and 15 vol.%. Additional annealing of these composites up to 500 K substantially decreases the percolation threshold down to below 10 vol.%. The electrical conductivity of composites with Ni@C inclusions close to the percolation threshold also sufficiently increases after additional annealing. However, with a concentration increase, the nanoparticles dispersion quality in the polymer is suppressed dramatically. This is proved by the SEM micrographs and the analysis of relaxation time distributions. It suggests that the additional improving of these composites is possible by the partial substitution of Ni@C with other nanoparticles, for example by CNT. For hybrid Ni@C/MWCNTs/epoxy composites series the electrical conductivity had a maximum close to 0.2 vol.% of Ni@C with absolute value several orders of magnitude higher than for sample with MWCNTs only. This indicates that a pronounced synergy effect between these two types of particles occurs at optimal Ni@C concentration. The dielectric relaxation spectroscopy allows estimating the nanoparticle distribution in the polymer matrix.

## Figures and Tables

**Table 1:** Parameters of the Arrhenius law fit for mono-filled Ni@C/epoxy composites.

Sample	Before Annealing			
	σ_0_, S/m	Error	E_A_/k_B_, K	Error
10 vol.% Ni@C	2.9 × 10^9^	1.8 × 10^9^	14,314	315
15 vol.% Ni@C	3.8 × 10^12^	2.0 × 10^12^	13,886	259
25 vol.% Ni@C	2.7 × 10^5^	1.1 × 10^5^	7579	202
30 vol.% Ni@C	2.2 × 10^6^	0.6 × 10^6^	7436	127
Pure epoxy	1.3 × 10^4^	0.4 × 10^4^	11,363	165

**Table 2:** Parameters of the Arrhenius law fit for hybrid Ni@C/MWCNTs/epoxy composites.

Sample	Before Annealing				After Annealing			
	σ_0_, S/m		E_A_/k_B_, K		σ_0_, S/m		E_A_/k_B_, K	
	Value	Error	Value	Error	Value	Error	Value	Error
0.09 vol.% MWCNT	4.1 × 10^2^	1.5 × 10^1^	9213	18	5.1 × 10^2^	9.9 × 10^1^	9284	101
0.09 vol.% MWCNT + 0.025 vol.% Ni@C	4.8 × 10^−4^	3.5 × 10^−5^	1532	35	5.7 × 10^−4^	1.3 × 10^−5^	1527	10
0.09 vol.% MWCNT + 0.2 vol.% Ni@C	1.8 × 10^−1^	0.7 × 10^−2^	3302	19	4.7 × 10^−3^	1.3 × 10^−4^	1308	12
0.09 vol.% MWCNT + 0.6 vol.% Ni@C	2.3 × 10^1^	2.0 × 10^1^	8049	450	4.0 × 10^−3^	2.9 × 10^−4^	3601	35
0.09 vol.% MWCNT + 1 vol.% Ni@C	1.9 × 10^5^	1.5 × 10^5^	12,634	393	7.1 × 10^−3^	4.4 × 10^−4^	3887	30

**Table 3:** Tunnelling model parameters for hybrid Ni@C/MWCNTs/epoxy composites.

Sample	σ_0_, S/m		T_1_, K		T_0_, K		T_1_/T_0_
	Value	Error	Value	Error	Value	Error	
0.09 vol.% MWCNT + 0.025 vol.% Ni@C	1.8 × 10^−5^	5.2 × 10^−8^	28.1	0.6	17.7	1.1	1.6
0.09 vol.% MWCNT + 0.2 vol.% Ni@C	2.5 × 10^−4^	7.1 × 10^−7^	28.1	0.5	4.5	0.7	6.2
0.09 vol.% MWCNT + 0.6 vol.% Ni@C	2.2 × 10^−6^	9.2 × 10^−9^	24.6	0.6	2.7	0.8	9.1
0.09 vol.% MWCNT + 1 vol.% Ni@C	2.7 × 10^−6^	3.4 × 10^−9^	18.5	0.2	3.8	0.5	4.9

## Data Availability

The data presented in this study are available on request from the corresponding authors.
